# Alcohol Misuse Link to POEMS Syndrome in a Patient

**DOI:** 10.3390/cancers9100129

**Published:** 2017-09-23

**Authors:** John Neary, Susan E. Goodwin, Lawrence B. Cohen, Manuela G. Neuman

**Affiliations:** 1 Department of Medicine, McMaster University, Hamilton, ON L8N 3Z5, Canada; john.neary@medportal.ca; 2 Juravinski Hospital, Hamilton, ON L8N 3Z5, Canada; fawcetts@mcmaster.ca; 3 Division of Gastroenterology, Sunnybrook Health Sciences Centre and Department of Internal Medicine, University of Toronto, Toronto, ON M4N 3M5, Canada; lawrence.cohen@sunnybrook.ca; 4 In Vitro Drug Safety and Biotechnology, Department of Pharmacology and Toxicology, University of Toronto, Toronto, ON M5G 1L5, Canada

**Keywords:** alcohol, malignancy, POEMS, biomarkers, VEGF, TNF-alpha, RANTES

## Abstract

Previously called Crow–Fukase syndrome, POEMS syndrome is characterized by poly-neuropathy, osteo-sclerotic myeloma, organomegaly, endocrinopathy, monoclonal plasma cell disorder, and skin changes. Extremely elevated levels of serum vascular endothelial growth factor (VEGF) are characteristic of the syndrome. Chronic hepatitis B (HBV) and C (HCV) infections can also be present in POEMS. The pathogenesis of the syndrome is not well understood. The link between chronic alcohol consumption and this malignant condition has not been reported until now. In addition, no previous study has evaluated the influence of cytokine and chemokines or viruses in the severity and evolution of POEMS. Objectives: (1) to describe a heavy-alcohol user, who was diagnosed with POEMS; (2) to demonstrate the utility of quantitative measurement of serum levels of VEGF in the diagnosis of POEMS and the monitoring of therapeutic interventions; (3) to demonstrate that overproduction of pro-inflammatory cytokines is a characteristic of POEMS. Methods: We describe a case of a POEMS patient presenting HCV and who is a heavy drinker; we compare the serum levels of cytokines and chemokines between the POEMS patient with 80 patients with HCV, 12 healthy controls, and 80 individuals with alcoholic liver disease (ALD). We quantified (ELISA pg/mL) the levels of VEGF, Interferon gamma (IFN-γ), Tumor Necrosis Factor alpha (TNF-α), Regulated-upon-Activation Normal-T-cell-Expressed and presumably-Secreted (RANTES), and Nuclear Factor kappa-B (NFκB). Results: In POEMS patients, VEGF levels were elevated versus control or other diseases, TNFα levels were higher versus control, but lower when compared with HCV or ALD patients. VEGF levels in POEMS patients decreased with therapeutic intervention. Conclusions: Chronic alcohol misuse can be a strong risk factor to rare malignancies such as POEMS syndrome. Extreme elevation of VEGF levels is diagnostic for POEMS syndrome, and should be followed to assess response to therapy. In addition, other comorbidities should be considered individually to ensure personalized therapeutic intervention.

## 1. Introduction

POEMS syndrome is a paraneoplastic syndrome due to an underlying plasma cell neoplasm. When making the diagnosis, it is important to consider that there are mandatory criteria, such as peripheral neuropathy (demyelinating 90%) and monoclonal gammopathy (lambda >90%). Important criteria are sclerotic bone lesions, elevated vascular endothelial growth factor, and the presence of Castleman disease [1,2]. Castleman disease—also known as giant or angiofollicular lymph node hyperplasia—lymphoid hamartoma, and angiofollicular lymph node hyperplasia, are a group of uncommon lymphoproliferative disorders that share common lymph node histological features that may be localized to a single lymph node (unicentric) or occur systemically (multicentric).

Multicentric Castleman disease (MCD) involves hyperactivation of the immune system, excessive release of proinflammatory chemicals (cytokines), proliferation of immune cells (B cells and T cells), and multiple organ system dysfunction [3]. Castleman disease must be distinguished from other disorders that can demonstrate “Castleman-like” lymph node features, including reactive lymph node hyperplasia, autoimmune disorders, and malignancies. While not officially considered a cancer, the overgrowth of lymphocytes with this disease is similar to lymphoma and more research is needed to search for small populations of neoplastic cells [1,2].

Minor features include organomegaly, endocrinopathy, characteristic skin changes, papilledema, extravascular volume overload, and thrombocytosis. POEMS syndrome is rare [1,2]. The diagnosis of POEMS syndrome is made with three of the major criteria, two of which must include polyradiculoneuropathy and clonal plasma cell disorder, and at least one of the minor criteria. High levels of vascular endothelial growth factor (VEGF) are the biomarker for diagnosis. VEGF elevation is the host’s immune response to neoplastic transformation of the plasma cells. The diagnosis does not require all the symptoms: peripheral neuropathy, organomegaly, endocrinopathy, monoclonal plasma cell disorder, skin changes. However, peripheral neuropathy (demyelinating 90%) and monoclonal gammopathy (lambda chain >90%) are diagnostic [1,2,3,4]. There are other symptoms equally important in POEMS that are not listed in the POEMS.

VEGF contributes to specific features of POEMS syndrome such as extravascular volume overload, organomegaly, and hemangioma [1,2,3,4,5,6,7,8,9,10]. Typical skin changes include hyperpigmentation, acrocyanosis, hemangioma, telangiectasia, hypertrichosis, and skin thickening. POEMS syndrome is chronic with a median survival greater than 5 years; however, the quality of life in these patients is poor because of progressive peripheral neuropathy [11,12,13,14,15,16]. The clinical manifestations of POEMS syndrome are, by definition, peripheral neuropathy and a monoclonal plasma cell disorder. The plasma cell always expresses lambda type light chains; the associated heavy chain is usually IgG or IgA, and rarely IgM. Virtually all patients have either solitary or multiple osteosclerotic bone lesions. The prevalence of other manifestations (e.g., organomegaly (spleen, liver, lymph nodes), endocrinopathy (pancreas, thyroid, gonadal, parathyroid, adrenal), skin changes, edema, hyperppigmentation, hypertrichosis, glomeruloid hemangiomata, plethora, acrocyanosis, flushing, white nails and papilledema) varies to a great extent. Additional findings associated with POEMS include extra-vascular fluid overload (edema, pleural effusion, or ascites), polycythemia, and fatigue. Moreover, clubbing, weight loss, hyperhidrosis, pulmonary hypertension/restrictive lung disease, and thrombotic diatheses may occur [14,15,16]. In addition to the clinical assessment, there are two general categories of noninvasive tests for POEMS: serologic panels of tests and radiologic tests. Serologic testing is more widely available. However, while tremendous progress has been made in improving the accuracy of serum markers of POEMS, only elevation of serum or plasma VEGF level is considered a diagnostic marker [6,17,18]. However, the cytokines and chemokines milieu may influence the manifestation of the disease and may be influenced by complications such as other diseases (alcoholic hepatitis, chronic viral hepatitis B and C).

We describe a case of POEMS syndrome that presented viral hepatitis C (HCV) infection and misused alcohol heavily. The patient was followed up clinically. In addition, the levels of cytokines and chemokines as markers of inflammation and repair were monitored for 4 years after the diagnosis of the disease. To understand further the possible mechanism of POEMS-induced damage, we examined a panel of cytokines in healthy individuals considered as normal controls, and individuals that without being diagnosed with POEMS syndrome, presented the same co-morbidities as our patient (e.g., alcohol abuse, HCV).

### Case Report

A 45-year-old man, Caucasian, was referred to an urgent internal medicine clinic in 2014 for assessment of recent onset peripheral edema and hematologic abnormalities. His medical history was notable for alcoholism (with a prior episode of pancreatitis), untreated chronic hepatitis C infection, depression, and vitamin B12 deficiency. Neuropathy, described as the sensation of “pins and needles”, was reported 1 year before presentation.

Nerve conduction studies in patients with POEMS syndrome show slowing of nerve conduction that is more predominant in the intermediate than distal nerve segment. The sensation in the knee and distal weakness were reduced. He presented normal cardiac, liver and renal function. He could not use a prosthesis due to edema of the leg. The skeletal survey was normal and the computed tomography for the chest, abdomen and pelvis showed splenomegaly, borderline lymphadenopathy.

An episode of necrotizing fasciitis leading to right below elbow amputation and left Symes amputation was also performed. He was living on the street and in shelters. His left foot prosthesis had broken several months previously. When he was assessed for a replacement prosthesis, he was noted to have peripheral edema that prevented prosthesis fitting. Routine laboratory investigations showed hypogonadism, polycythemia, thrombocytosis, and neutrophilia. In the internal medicine clinic, the patient complained of paraesthesias, subjective sensory loss, and a tingling, burning pain in his distal extremities. Moderate peripheral edema and hepatosplenomegaly were found on examination. Abdominal ultrasound showed hepatosplenomegaly with the spleen measuring 13.4 cm. The patient did not have a biopsy or a Fibroscan to determine his fibrosis status. Bilirubin levels were in the normal range for the entire period of time. Alanine aminotransferase (ALT) fluctuated 26–30 IU, and aspartate aminotransferase (AST) fluctuated 36–39 IU. Hepatitis C viral load was 2.1 × 10^6^.

An echocardiogram revealed normal biventricular function. The skeletal survey was normal.

Laboratory investigations were remarkable for hemoglobin 190g/L, platelets 687 × 10^9^/L, neutrophils 37.4 × 10^9^/L, total testosterone 2.2 nmol/L (normal range, 5.5–25.2), and prolactin 24.3 mcg/L (<15). Blood film examination showed normal morphology. Electrolytes, creatinine, alanine and aspartate aminotransferases, bilirubin, C-reactive protein, and urinalysis were normal. Serum protein electrophoresis demonstrated polyclonal gammopathy. Analysis of peripheral blood for JAK2 mutation and BCR-ABL fusion product was negative. The immunofixation-revealed <1 gm/L amount of lambda light chain.

POEMS syndrome was considered despite the normal results of the serum protein electrophoresis. Computed tomography of the chest, abdomen, and pelvis confirmed hepatosplenomegaly and showed prominent retroperitoneal lymph nodes. Electromyography showed a demyelinating polyneuropathy. Lumbar puncture was performed; albumin-cytologic dissociation was seen on analysis of cerebrospinal fluid. Bone marrow aspiration and biopsy revealed hypercellular marrow with 5% plasma cells.

Serum protein electrophoresis was repeated, again showing polyclonal gammopathy; the kappa/lambda ratio in peripheral blood was normal. Serum protein electrophoresis was repeated with immunofixation and instructions to look carefully for a low-titer monoclonal protein. An IgA M-protein with lambda light chain was identified, with a titer of less than 1 g/L. A 24-h urine collection for protein electrophoresis was not feasible given the patient’s homelessness and mobility impairment.

VEGF level was markedly elevated at 2800 pg/mL (normal 30–90). Due to the diagnostic value of VEGF, the patient was referred to the malignant hematology clinic with a new diagnosis of POEMS syndrome. The treatment with dexamethasone and lenalidomide was initiated. Eight months later, his edema and neuropathic symptoms had improved somewhat and his VEGF level had decreased to 1828 pg/mL despite some non-adherence with treatment. At 17 months from diagnosis, his VEGF level had further decreased to 1022 pg/mL, although his edema and symptoms of polyneuropathy did not show further improvement.

Stem cell harvest was performed at 20 months from diagnosis. At 22 months from diagnosis, he was admitted to hospital with bilateral infected leg ulcers. He improved quickly with antibiotics but refused to restart dexamethasone and lenalidomide. His VEGF level fell to 540 pg/mL at 29 months from diagnosis despite a 7-month gap in treatment; however, his peripheral edema had worsened to the point that his left foot prosthesis once again did not fit, and his neuropathic pain had become less responsive to analgesics. At this point, he was not taking his medication. In addition, he also began drinking heavily. This fact led to a remission of POEMS. The levels of VEGF increased to 3480 pg/mL. This fact clearly demonstrates the link between the clinical and laboratory manifestation of POEMS syndrome and alcohol misuse.

## 2. Laboratory Methods

Tumor necrosis factor (TNF-α) levels (pg/mL) were quantitatively determined in serum, using enzyme-linked immunosorbent-assay (ELISA). Cytokine and chemokine levels were measured by ELISA as follows: TNFα (R&D Systems Inc., Minneapolis, MN, USA), IL-1, IL-8, VEGF (PeproTech, NY, USA); IL-6, Regulated upon Activation, Normal T cell Expressed and presumably Secreted RANTES; (eBioScience, Frederick, MI, USA) with 96% sensitivity and 92% specificity. NF-κBp65 activity was measured by ELISA (InVitrogen Corporation, Camarillo, CA, USA).

RANTES plays a primary role in the inflammatory immune response via its ability to chemo-attract leukocytes and modulate their function. Aliquots from the sample were added to the 96-well plate. Each specimen was analyzed in duplicate with 95% sensitivity and 92% specificity. Our measurement system demonstrates strong correlations across replicates with correlation coefficients >0.99, ensuring reliable detection of differences in cytokine levels between biological samples. We used standards and reference reagents available from the National Institute for Biological Standards and Controls (NIBSC, Herts, UK). These methods are standardized in our laboratory according to the procedures described.

To determine the HCV viral load, we employed the automated, sensitive method COBAS AmpliPrep/COBAS TaqMan^®^ HCV Test (Roche Diagnostics, Quebec, PQ, Canada). The lower limit of detection is 15 IU/mL (1 IU/mL × 2.7 copies/IU = copies/mL).

## 3. Results

**Figure 1:**
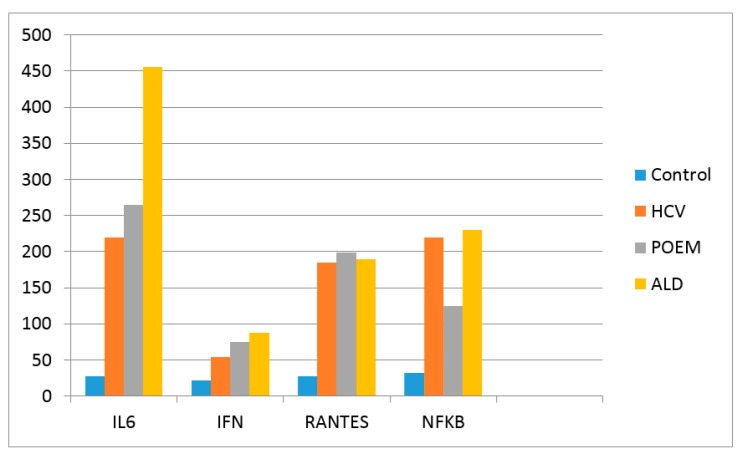
Levels of Interleukin 6, INFγ, RANTES and NFκB. The values are presented as pg/mL. The results of the 12 control individuals and patients with HCV (80) and ALD (80) represent the median value of the group. HCV patients were male and female Caucasians with a median age of 39, non-cirrhotic with a fibrosis score F0–F1. ALD patients were only Caucasian male with a median age of 52, non-cirrhotic with a fibrosis score F0–F1. All these patients have been evaluated in a Hepatology clinic.

**Figure 2:**
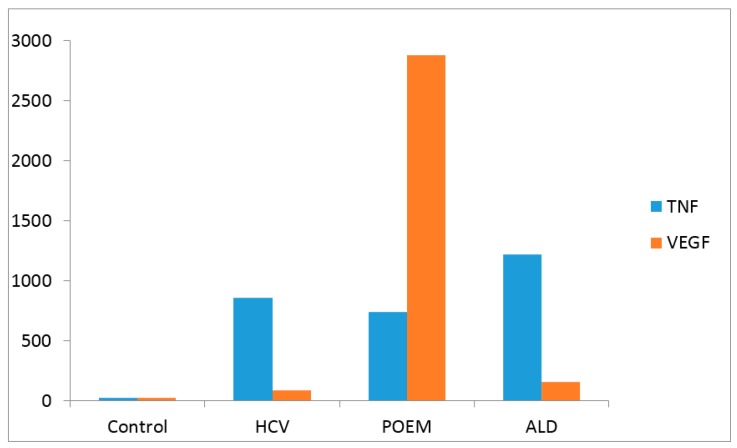
Levels of TNF-α and VEGF (pg/mL). The POEMS + ALD + HCV patients were compared with 12 healthy controls, 80 patients with HCV, and 80 with ALD. Results are given in pg/mL. The results of the 12 control individuals and patients with HCV (80) and ALD (80) represent the median value of the group.

Table 1 and Figure 1 and Figure 2 present the laboratory data in the patient compared to other individuals that have been studied in our laboratory using the same methodology, standards and controls. The eight patients diagnosed with POEMS syndrome were male and female presenting VEGF 2876 ± 714 pg/mL. They did not have HCV and were not known as heavy drinkers.

HCV patients were male and female Caucasians with a median age of 39, non-cirrhotic with a fibrosis score F0–F1. Patients presenting ALD were only Caucasian males with a median age of 52, non-cirrhotic with a fibrosis score F0–F1. All these patients have been evaluated in a Hepathology clinic.

There is a statically significant difference between the levels of cytokines in control and the diseased patients (*p* < 0.001). The ALD patients presented statistically higher levels of interleukins IL-8 and IL 18 (*p* < 0.05) when compared with POEMS and HCV patients, but lower IL 12 when compared with the HCV patients. The levels of these interleukins in the patient described in this paper (POEMS + ALD + HCV) were in the range of the POEMS patients (Table 1).

Figure 1 shows that there are statistically higher levels of serum IL-6 in ALD individuals when compared with HCV. The level of IL-6 in POEMS + ALD + HCV patient was in the range of the HCV patients.

Figure 2 shows that the level of TNF-α in our patients was in the range of the TNF in HCV-infected individuals. However, the VEGF level in this individual was extremely elevated.

## 4. Discussion

POEMS syndrome is a paraneoplastic disorder secondary to an underlying plasma cell dyscrasia. The POEMS syndrome patient described in this article displayed polyneuropathy and monoclonal plasma cell disorder. VEGF elevation was used for diagnosis [17,18].

POEMS syndrome’s pathogeny is complex and remains unclear. Diverse studies have stressed that patients present high levels of proangiogenic and proinflammatory cytokines such as IL-1β, TNF-α, or IL-6. These interleukins stimulate production of-secreted VEGF, which has been found to be high in many POEMS patients. This factor, as its name indicates, targets endothelial cells, inducing cell proliferation and increased capillary permeability responsible for most of the syndrome’s characteristic manifestations. Several researchers have proposed that there may be a close relation between IL-6 levels, VEGF, and disease activity [11,12,19,20,21].

A variety of serologic markers have been evaluated to predict the degree of inflammation and fibrosis in the liver, and panels have been developed that combine assays of multiple markers to improve predictive ability. However, the scope of the present paper is to diagnose and monitor the inflammation and possible repair of a POEMS syndrome patient, who has co-morbidities (e.g., ALD and HCV). The patient was followed by an internal medicine specialist and neurologist. The patient was not seen by a hepatologist for treatment of the liver injury.

Most POEMS patients receive corticosteroids, intravenous gammaglobulin, plasmapheresis, chemotherapy (cyclophosphamide or high-dose melphalan) or radiation therapy. Novel myeloma therapies include thalidomide, lenalidomide and anti-VEGF antibodies (bevacizamib). Regarding the therapeutic intervention in this patient, the decision was taken in concordance with the present recommendations, i.e., lenalidomide with autologous peripheral blood stem cell transplant (ASCT) [22,23,24,25,26,27].

## 5. Conclusions

The present communication is the first to report the link between alcohol misuse and the presence of haematologic malignancy with neurologic–dermatologic consequences. The case report described an effective laboratory panel as a diagnostic tool for patients with this condition. The level of chemokines and cytokines including VEGF and TNF may show the need for specific therapeutic approaches. Moreover, the level of cytokines and chemokines monitored over time can guide step-by-step monitoring of disease status.

In this context, the present case report coincides with effective therapies for POEMS syndrome in which the clinical observations, clinical intuition and laboratory diagnosis cooperate successfully. The main role of the laboratory diagnostic is to confirm the clinical diagnosis by providing the VEGF levels. In addition, other cytokines tests can provide complementary information in patients with indeterminate gamma-pathologic results or results that are at variance with the clinical impression.

The POEMS + ALD + HCV patient was compared with 12 healthy Caucasian controls, 80 patients with HCV, 8 with POEMS and 80 with ALD. Results are given in pg/mL. HCV patients were male and female Caucasians with a median age of 39, non-cirrhotic with a fibrosis score F0–F1. ALD patients were only Caucasian males with a median age of 52, non-cirrhotic with a fibrosis score F0–F1.

## Figures and Tables

**Table 1:** Levels of interleukins.

Disease	IL 1β	IL-8	IL 12	IL 18
Control	12 ± 2	20 ± 4	22 ± 4	22 ± 6
ALD	104 ± 12	876 ± 14	98 ± 8	218 ± 12
HCV	44 ± 6	245 ± 15	232 ± 16	154 ± 14
POEMS + ALD + HCV	56	68	94	175
POEMS	74 ± 22	46 ± 24	98 ± 12	182 ± 16

## Abbreviations

ALD	alcoholic liver disease
ALT	alanine amino transferase
AST	aspartate aminotransferase
HCV	hepatitis C virus infection
IL	interleukin
IFN-γ	Interferon gamma
NFκB	Nuclear Factor kappa-B
POEMS	peripheral neuropathy, organomegaly, endocrinopathy, monoclonal plasma cell disorder, skin changes
RANTES	Regulated-upon-Activation Normal-T-cell-Expressed and presumably-Secreted
TNF	tumor necrosis factor
VEGF	vascular endothelial growth factor
